# Orai1- and STIM1-mediated calcium signaling controls PD-L1 expression and modulates antitumor immunity in oral cancer

**DOI:** 10.1016/j.jphyss.2026.100063

**Published:** 2026-02-03

**Authors:** Eriko Yamashita, Soichiro Ishikawa, Yuto Mizuno, Yu Iida, Mio Mochizuki, Akane Nagasako, Michiko Endo, Kohei Osawa, Yayoi Kimura, Takayuki Fujita, Utako Yokoyama, Kenji Mitsudo, Yoshihiro Ishikawa, Masanari Umemura

**Affiliations:** a Cardiovascular Research Institute, Yokohama City University Graduate School of Medicine, 3-9 Fukuura, Kanazawa-ku, Yokohama-shi, Kanagawa 236-0004, Japan; b Department of Oral and Maxillofacial Surgery, Yokohama City University Graduate School of Medicine, 3-9 Fukuura, Kanazawa-ku, Yokohama-shi, Kanagawa 236-0004, Japan; c Department of Clinical Oral Oncology, National hospital organization, Hokkaido Cancer Center, 3-54, 2-chome, Kikusui 4-jo, Shiroishi-ku, Sapporo-shi, Hokkaido 003-0804, Japan; d Department of Environmental Immuno-Dermatology, Yokohama City University Graduate School of Medicine, 3-9 Fukuura, Kanazawa-ku, Yokohama-shi, Kanagawa 236-0004, Japan; e Department of Neurosurgery, Yokohama City University Graduate School of Medicine, 3-9 Fukuura, Kanazawa-ku, Yokohama-shi, Kanagawa 236-0004, Japan; f Department of Oral and Maxillofacial Surgery/Orthodontics, Yokohama City University Medical Center, 4-57 Urafune-cho, Minami-ku, Yokohama-shi, Kanagawa 232-0024, Japan; g Advanced Medical Research Center, Yokohama City University Graduate School of Medicine, 3-9 Fukuura, Kanazawa-ku, Yokohama-shi, Kanagawa 236-0004, Japan; h Department of Physiology, Fukuoka University, 7-45-1 Nanakuma, Jonan-ku, Fukuoka-shi, Fukuoka 814-0180, Japan; i Department of Physiology, Tokyo Medical University, 1-5-45 Yushima, Bunkyo-ku, Tokyo 113-8510, Japan; j Yokohama City University President, 3-9 Fukuura, Kanazawa-ku, Yokohama-shi, Kanagawa 236-0004, Japan

**Keywords:** Store-operated calcium entry (SOCE), PD-L1, Oral squamous cell carcinoma (OSCC), Interferon-gamma (IFN-γ), Tumor immune evasion

## Abstract

This study investigated the regulation of PD-L1 expression by calcium signaling in oral squamous cell carcinoma (OSCC). We found that IFN-γ stimulation markedly altered the intracellular proteome and activated calcium-associated pathways. Pharmacological inhibition of store-operated calcium entry (SOCE) using the Orai1 inhibitor Synta66 suppressed IFN-γ-induced PD-L1 expression in a dose-dependent manner. Consistently, knockdown of Orai1 or STIM1 attenuated PD-L1 induction under IFN-γ stimulation, while basal expression remained unchanged. Inhibition of CaMK2 and CaMKK2 also reduced PD-L1 expression, indicating involvement of calmodulin-dependent kinase pathways. Functionally, Orai1 or STIM1 knockdown enhanced CD8⁺ T cell–mediated suppression of OSCC cell proliferation. Collectively, these results demonstrate that SOCE-mediated calcium signaling and downstream kinases regulate IFN-γ-induced PD-L1 expression and suggest that targeting SOCE components could represent a novel therapeutic approach to overcoming tumor immune evasion in OSCC. (132 words)

## Highlights

• IFN-γ reshapes the proteome and activates calcium pathways in oral squamous carcinoma.
• SOCE and CaM-dependent kinases control IFN-γ–induced PD-L1 expression in OSCC.
• Targeting SOCE boosts CD8⁺ T cell suppression of OSCC, offering strategy to block immune evasion.

## Introduction

Oral squamous cell carcinoma (OSCC), a subset of head and neck cancers, refers to malignant tumors arising in the oral cavity. OSCC accounts for approximately 2 % of all cancers worldwide, and its incidence has been increasing in recent years [1]. While advances in surgical techniques have improved overall survival, prognosis remains poor for patients with advanced, recurrent, or metastatic disease. In this context, immune checkpoint inhibitors (ICIs) targeting the Programmed cell death protein 1 (PD-1)/Programmed cell death ligand 1 (PD-L1) axis have emerged as a promising therapeutic option. Since 2016, anti-PD-1 antibody therapy has been approved for platinum-resistant recurrent or metastatic head and neck cancers, marking a new era in cancer immunotherapy. PD-L1, expressed on tumor cells, binds to PD-1 receptors on cytotoxic T cells, thereby suppressing antitumor immunity and enabling immune evasion. PD-L1 expression is known to be induced by interferon-gamma (IFN-γ) stimulation [2], [3]. However, the molecular mechanisms governing this regulation are not fully elucidated. In our recent study in melanoma, we demonstrated that Focal adhesion kinase and its homolog Proline-rich tyrosine kinase 2 exert distinct roles in IFN-γ–mediated PD-L1 upregulation and immune checkpoint resistance [4]. Building on these findings, the present study extends this line of research to OSCC, aiming to further elucidate the intracellular signaling pathways that regulate IFN-γ–induced PD-L1 expression.

ICIs, such as anti-PD-1 antibodies (e.g., nivolumab), have been reported to exert certain therapeutic efficacy in patients with recurrent or metastatic head and neck squamous cell carcinoma and are increasingly being applied to oral cancer [2], [3]. However, several clinical challenges have been noted. First, the objective response rate to anti-PD-1 monotherapy remains limited, at approximately 13–18 %, leaving many patients without clinical benefit [5]. Additionally, even in patients who initially respond, the acquisition of resistance during treatment has been reported, leading to attenuation or loss of therapeutic efficacy [6]. Although biomarkers such as PD-L1 expression levels and tumor mutational burden have been utilized as indicators of treatment responsiveness, accurately predicting the therapeutic outcomes remains difficult [7]. Furthermore, immune-related adverse events caused by autoimmune reactions, including interstitial pneumonitis, endocrine disorders, and colitis, occur with a certain frequency, sometimes necessitating treatment discontinuation [8]. The high cost of anti-PD-1 antibodies also imposes a significant economic burden, particularly when long-term administration or combination therapies are required [9]. Moreover, oral cancer possesses a unique immune microenvironment that is constantly exposed to commensal microbiota and chronic inflammation, unlike other types of cancer, which may contribute to the unstable efficacy of ICIs [10]. Considering these challenges, elucidation of novel molecular mechanisms and the development of effective combination strategies are needed to enhance the therapeutic efficacy of PD-1/PD-L1-targeted therapies. Therefore, investigating the intracellular signaling pathways that regulate PD-L1 expression may provide new insights for improving the efficacy of cancer immunotherapy. In this regard, recent studies have suggested a potential role for intracellular calcium signaling in modulating PD-L1 expression in cancer cells [11].

Recent studies have indicated that intracellular Ca²⁺ signaling plays a role in the transcriptional regulation of PD-L1 [12]. In non-excitable cells, store-operated calcium entry (SOCE) is the primary mechanism for Ca²⁺ influx, triggered by the depletion of endoplasmic reticulum (ER) Ca²⁺ stores. Upon ER store depletion, the ER-resident calcium sensor STIM1 (Stromal Interaction Molecule 1) becomes activated and interacts with Orai1, a plasma membrane calcium channel, to mediate Ca²⁺ entry into the cytoplasm [13]. Orai1 is thus a critical component of the SOCE machinery [14].

Our previous work has demonstrated that SOCE contributes to the proliferation and migration of melanoma cells [15]. Additionally, dysregulation of SOCE has been shown to promote tumor progression by altering calcium homeostasis, enhancing cell motility and invasiveness [16]. Although SOCE is known to be involved in various cellular functions, its role in regulating tumor immune responses—particularly PD-L1 expression—remains largely unexplored [17]. Orai1 is the pore-forming subunit of the calcium release-activated calcium (CRAC) channel and was first identified in 2006 as the gene mutated in patients with severe combined immunodeficiency [14]. Based on these findings, we hypothesized that SOCE may be critically involved in the regulation of IFN-γ-induced PD-L1 expression in OSCC cells. The aim of this study was to elucidate the SOCE-dependent mechanism of PD-L1 regulation in oral cancer, with a view to identifying novel molecular targets for cancer immunotherapy.

## Material and methods

### Reagents

Human IFN-γ was purchased from FUJIFILM Wako Pure Chemical Corporation (Osaka, Japan). The SOCE channel inhibitor Synta66, which targets Orai1 forming the pore of the CRAC channel, was purchased from MedChemExpress (Monmouth Junction, NJ, USA), respectively. The CaMK2 inhibitor KN-62 was purchased from Sigma Aldrich (St. Louis, MO, USA), respectively. KN-62 is a CaMK2 inhibitor that non-selectively targets multiple CaMK2 isoforms (α, β, γ, and δ) by interfering with Ca²⁺/calmodulin binding and was therefore used as an inhibitor of total CaMK2 activity. The CaMKK2 inhibitor STO-609 was purchased from Cayman Chemical (Ann Arbor, MI, USA). Recombinant human IL-2 was obtained from PeproTech (Cranbury, NJ, USA). All reagents were used at specified concentrations in the experiments.

## Cell lines

Human oral squamous cell carcinoma cell lines HSC-3 and OSC-19 were purchased from Health Science Research Resources Bank (Japan Health Sciences Foundation) [18], [19], [20].

HSC-3 and OSC-19 cells were cultured in Dulbecco’s Modified Eagle Medium (Sigma Aldrich) supplemented with 10 % fetal bovine serum (BioWest, Riverside, MO, USA).

## Proteomic analysis

HSC-3 human oral squamous cell carcinoma cells (shCTRL) were seeded at a density of 5.0 × 10⁵ cells per well and cultured at 37 ℃ in a humidified 5 % CO₂ atmosphere until 80–90 % confluence. Cells were treated with or without interferon-γ (IFN-γ; 100 ng/mL) for 6 h. Four biological replicates (n = 4) were prepared for each condition. After treatment, cells were washed 2–3 times with cold phosphate-buffered saline (PBS) and lysed in 0.1 mL of lysis buffer (50 mM NH₄HCO₃, 8 M urea) per well. The lysate was sonicated on ice and centrifuged at 15,000 × g for 10 min at 4 ℃, and the supernatant was collected. Protein concentration was determined using the Bicinchoninic acid assay.

A total of 50 μg of protein per sample was reduced with dithiothreitol (DTT; final concentration 10 mM) at 37 ℃ for 60 min, followed by alkylation with iodoacetamide (final concentration 25 mM) for 30 min in the dark at room temperature. The samples were diluted fourfold with 50 mM NH₄HCO₃ to reduce the urea concentration to below 2 M. Trypsin (Promega) was added at an enzyme-to-substrate ratio of 1:20, and digestion was performed at 37 ℃ overnight (∼16 h). After desalting using C18 StageTips [21], peptides were lyophilized and stored at −80 ℃ until analysis. For LC-MS/MS analysis, 10 μg of peptides were dried and reconstituted, and 0.5 μg equivalent was injected per run.　LC-MS analysis of eluted samples was performed using a QExactive HF mass spectrometer (Thermo Fisher Scientific) coupled to an UltiMate 3000 LC system (Thermo Fisher Scientific). The analytical conditions were as follows: Nano HPLC capillary column, 75 μm × 180 mm (C18, 3 μm; Nikkyo Technos, Tokyo, Japan); mobile phase, (A) 0.1 % formic acid / 2 % ACN, (B) 0.1 % formic acid / 95 % ACN; gradient, 115 min at a flow rate of 300 nL/min; polarity, positive; dynamic exclusion, auto; NCE, 27; scan range, 300–1500 *m/z*; top N, 20. To identify peptides, peak lists were created using Proteome Discoverer software (version 2.2; Thermo Fisher Scientific).　Protein identification was performed using the MASCOT search engine (version 3.0; Matrix Science, London, UK). Mascot search parameters were as follows: database, human protein sequences (20,435 sequences) in the UniProt Knowledgebase database released in June 2024; enzyme, trypsin; peptide mass tolerance, ±5 ppm; fragment mass tolerance, ±0.05 Da; maximum missed cleavages, 2; and variable modifications, acetyl (protein N-term), oxidation (M), carbamyl (N-term), and carbamidomethyl (C). A false discovery rate of less than 1 % and a peptide score ≥ 30 was adopted as the acceptance criteria for identifications. For proteomic experiments, four independently prepared samples per condition (n = 4 biological replicates) were analyzed by LC–MS/MS (one technical replicate per sample). False discovery rates (FDRs) were controlled at < 1 % at both the peptide and protein levels.

## Proteomic data analysis

Protein expression data were obtained from label-free quantification of total proteomes. Differentially expressed proteins were identified based on log₂ (fold change) > 0.58 or < −0.58 and *p*-value < 0.05. Volcano plots and heatmaps were generated using R version 4.3.2. Functional enrichment analysis was performed using Metascape, with a relaxed threshold of log₂ (fold change) > 0.4 or < −0.4 and *p*-value < 0.05 [22]. Network visualization of enriched terms was also conducted using Metascape.

## Data and software availability

All mass spectrometry proteomics data were deposited in the ProteomeXchange Consortium (http://www.proteomexchange.org) via the jPOST (https://jpostdb.org) partner repository, with the dataset identifier PXD066968 (https://repository.jpostdb.org/preview/15501308716891bfa8243bd), Access key: 2685. All data were available without restrictions [23].

## Western blot

Western blot analyses were performed as previously described [15], [24], [25]. The primary antibodies used were as follows:

- Anti-PD-L1 (Cell Signaling Technology, MA, USA) at 1:1000 dilution.

- Anti-Orai1 (Sigma-Aldrich) at 1:1000 dilution

- Anti-STIM1 (Cell Signaling Technology) at 1:1000 dilution

- Anti-GAPDH (Santa Cruz Biotechnology, CA, USA) at 1:4000 dilution (used as a loading control)

-Anti-phospho-CaMK2 (Cell Signaling Technology, MA, USA) at 1:1000 dilution.

-Anti-phospho-CaMKK2 (Cell Signaling Technology, MA, USA) at 1:1000 dilution.

Western blot analyses were performed using three or four independently prepared biological replicates (n = 3–4), depending on the experiment. Representative blots from independent cultures are shown.

## Cell proliferation assay

Cell proliferation was assessed using the Cell Counting Kit-8 (CCK-8) assay (Dojindo, Kumamoto, Japan) [26]. Cells were seeded in 96-well plates, and absorbance was measured at 450 nm to calculate the cell proliferation rate.

## Short hairpin RNA transduction

HSC-3 cells were subjected to transduction with Orai1 shRNA, STIM1 shRNA and scramble control shRNA. Transductions with lentivirus Sigma-Aldrich and Santa Cruz Biotechnology were carried out as previously described [15], [25].

## Quantitative real-time reverse transcription–polymerase chain reaction

Total RNA was extracted using ISOSPIN Cell & Tissue RNA kit (Nippon Gene, Tokyo, Japan) [27]. cDNA was synthesized with PrimeScript RT Reagent Kit (TaKaRa Bio, Shiga, Japan), and qRT-PCR was performed on a StepOnePlus Real-Time PCR System (Applied Biosystems, Waltham, MA, USA). Relative gene expression was calculated by the 2^-ΔΔCT method with 18S rRNA as the internal control. Primer sequences for human genes were as follows:

- CD274 (PD-L1):

- Forward: 5’-GCTGCACTAATTGTCTATTGGGA-3’

- Reverse: 5’-AATTCGCTTGTAGTCGGCACC-3’

- Orai1:

- Forward: 5’-GACTGGATCGGCCAGAGTTAC-3’

- Reverse: 5’-GTCCGGCTGGAGGCTTTAAG-3’

- STIM1:

- Forward: 5’-CACTCTTTGGCACCTTCCACGT-3’

- Reverse: 5’-CTGTCACCTCGCTCAGTGCTTG-3’

- 18S rRNA (normalization control):

- Forward: 5’-ATGCCCATCACTCGGATGC-3’

- Reverse: 5’-CCCTGCTTTGTATCGGCCTG-3’

## Fluorescence imaging of intracellular calcium

Live-cell calcium imaging was performed as previously described with some modifications. SOCE was assessed by live-cell Ca²⁺ imaging using the fluorescent Ca²⁺ indicator Fluo 4-AM (Dojindo) [23]. HSC-3 cells were loaded with Fluo 4-AM (final concentration, 5 µM) in HBSS containing Ca²⁺ and Mg²⁺ (HBSS (+) without Phenol Red; FUJIFILM Wako Pure Chemical Corporation) supplemented with Pluronic F-127 (Sigma-Aldrich) for 30 min at 37 ℃ in the dark. After dye loading, cells were washed with recording buffer and allowed to equilibrate before imaging.

For pharmacological inhibition experiments, cells were pretreated with the SOCE inhibitor Synta66 (20 µM) for 3 h prior to imaging. For genetic inhibition experiments, shCTRL, shOrai1, or shSTIM1 cells were analyzed as indicated.

Live-cell Ca²⁺ imaging was initiated under Ca²⁺-free conditions using HBSS without Ca²⁺ and Mg²⁺ (HBSS (-) without Phenol Red) supplemented with EGTA (Dojindo). After acquisition of baseline fluorescence, endoplasmic reticulum Ca²⁺ stores were depleted by the addition of thapsigargin (FUJIFILM Wako Pure Chemical Corporation) at 120 s. Extracellular Ca²⁺ was subsequently reintroduced by switching to HBSS containing Ca²⁺ and Mg²⁺ (HBSS (+) without Phenol Red) at 420 s to evoke SOCE. Fluorescence images were acquired continuously throughout the imaging period.

Fluorescence intensity was quantified by defining individual regions of interest (ROIs) from 50 randomly selected cells per condition. Baseline fluorescence (F₀) was defined as the fluorescence intensity immediately before thapsigargin addition, and changes in intracellular Ca²⁺ levels were expressed as ΔF/F₀, calculated as (F−F₀)/F₀. Data are presented as the mean ± standard error of the mean (SEM) of individual ROIs.

This experiment was performed to validate SOCE inhibition under the indicated pharmacological and genetic conditions, and representative Ca²⁺ traces are shown. All data processing and graphical representations were performed using R (version 4.5.2) with RStudio.

## Cytotoxicity assay (Killing Assay)

Peripheral blood mononuclear cells (PBMCs) were isolated from healthy donors using SepMate™ (STEMCELL Technologies, Cat# ST-0238). CD8⁺ T cells were subsequently purified from PBMCs using the EasySep™ CD8 Positive Selection Kit II (STEMCELL Technologies, Vancouver, BC, Canada, Cat# ST-0186) with the EasySep™ Magnet and Buffer, according to the manufacturer’s instructions. Purified CD8⁺ T cells were expanded in ImmunoCult™-XF T Cell Expansion Medium (STEMCELL Technologies, Cat# 10981) supplemented with ImmunoCult™ Human CD3/CD28 T Cell Activator (STEMCELL Technologies, Cat# ST-10971, ST-10991) and recombinant human IL-2 (100 U/mL; PeproTech). Target cancer cells (control, Orai1 knockdown, and STIM1 knockdown) were seeded into E-plates and allowed to adhere. When the cell index reached approximately 1.0, the culture medium was replaced with fresh medium, and CD8⁺ T cells were added at an effector-to-target (E:T) ratio of 20:1. Tumor cell proliferation was then monitored in real time using the xCELLigence Real-Time Cell Analysis System (ACEA Biosciences, San Diego, CA, USA) to evaluate cytotoxic activity [4], [28].

## Flow cytometry

PBMCs were isolated from healthy donors by density gradient centrifugation. Cells were stained with fluorescence-conjugated antibodies against human CD4 (FITC) and CD8 (PE-Cy7) (BD Pharmingen, San Diego, CA, USA), together with 7-AAD (BD Biosciences, San Jose, CA, USA) for viability as previously described [29]. Isotype controls (FITC- and PE-Cy7-conjugated mouse IgG1 κ) were included to confirm specificity. Compensation was performed with unstained and single-stained controls. After staining, cells were analyzed on a BD FACSCelesta™ flow cytometer, and data were processed with FlowJo software (v10). Dead cells (7-AAD⁺) were excluded, lymphocytes were gated by forward/side scatter, and CD4/CD8 expression was analyzed using quadrant gating. Experiments were repeated at least three times.

## Data analysis and statistics

Statistical analysis was performed using GraphPad Prism 9 software (GraphPad Software Inc., San Diego, CA, USA). After confirming that the data followed a normal distribution, a two-tailed Student’s t test was used to determine the significance of differences between two independent groups. Pearson’s correlation analysis was performed to assess correlations between two variables. Comparisons among more than two groups were performed using one-way analysis of variance (ANOVA) followed by Tukey’s post hoc test. Quantitative data are presented as mean ± SEM. Exact p-values or significance levels (p < 0.001 or ns, not significant) are indicated in the figures, and p < 0.05 was considered statistically significant.

## Results

### IFN-γ stimulation alters the proteome and activates calcium-related pathways

**Fig. 1:**
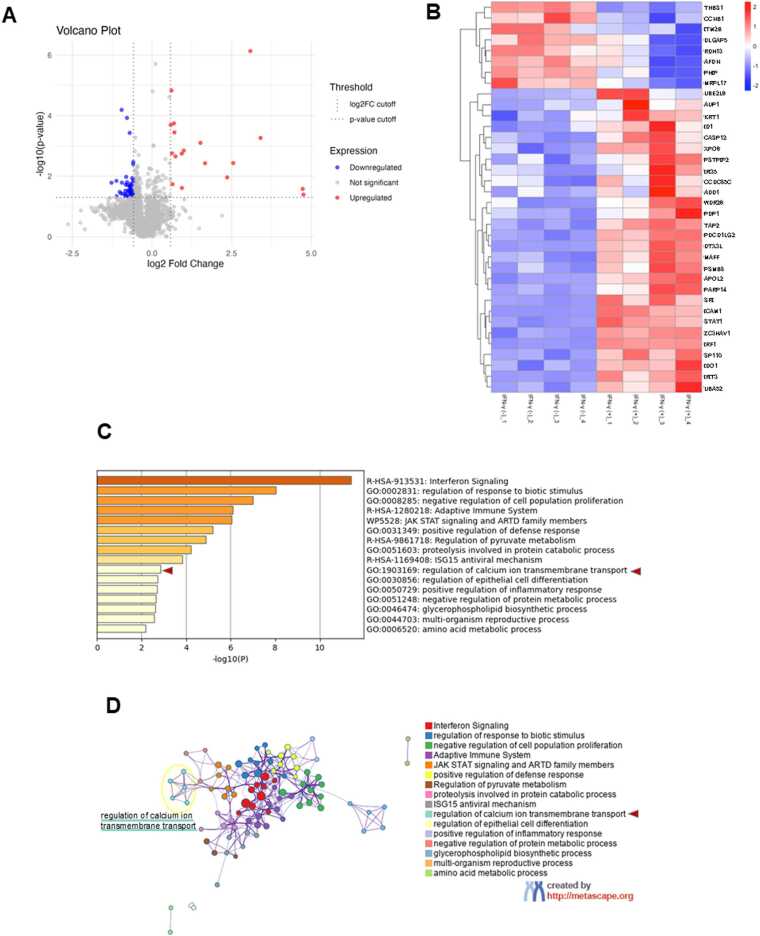
Proteomic profiling reveals calcium-related pathway enrichment following IFN-γ stimulation in OSCC cells. (A) Volcano plot of protein expression changes after IFN-γ stimulation. Red and blue dots indicate significantly up- and downregulated proteins (log₂FC > 0.58 or < −0.58, p < 0.05). (B) Heatmap of significantly altered proteins (same thresholds as in A) in HSC-3 cells with and without IFN-γ treatment. Red and blue indicate increased and decreased expression, respectively. (C) Enrichment analysis of differentially expressed proteins (log₂FC > 0.4 or < −0.4, p < 0.05) using Metascape. Calcium ion transport–related GO terms were significantly enriched (highlighted). (D) Network visualization of enriched functional clusters using Metascape. Calcium ion transport–related terms were grouped with immune-associated pathways, including interferon signaling. Node size reflects significance.

IFN-γ is a representative cytokine that induces antitumor immune responses and is known to regulate PD-L1 expression in tumor cells. To comprehensively investigate the signaling mechanisms involved in PD-L1 regulation, we performed proteomic profiling of the human OSCC cell line HSC-3 under IFN-γ-stimulated and unstimulated conditions. Volcano plot analysis revealed significant expression changes in 36 proteins upon IFN-γ stimulation (Fig. 1A). Hierarchical clustering of the normalized abundances of significantly changed proteins in the heatmap analysis demonstrated distinct expression patterns between the stimulated and unstimulated groups, indicating stimulus-dependent proteomic alterations (Fig. 1B). Enrichment analysis of the differentially expressed proteins identified significant enrichment of pathways and processes related to immune and antiviral responses, as expected. Notably, calcium-related Gene Ontology (GO) terms such as "regulation of calcium ion transmembrane transport" were also significantly enriched (Fig. 1C). Furthermore, network visualization demonstrated that calcium-related cluster, together with immune-associated pathways including interferon signaling, were enriched following IFN-γ stimulation and spatially grouped within the network (Fig. 1D).

The distinct clustering of calcium-related GO terms observed in response to IFN-γ stimulation prompted further investigation into the potential involvement of calcium signaling in the regulation of PD-L1 expression and other tumor immune-related factors.

## SOCE inhibition suppresses IFN-γ-induced PD-L1 mRNA and protein expression

**Fig. 2:**
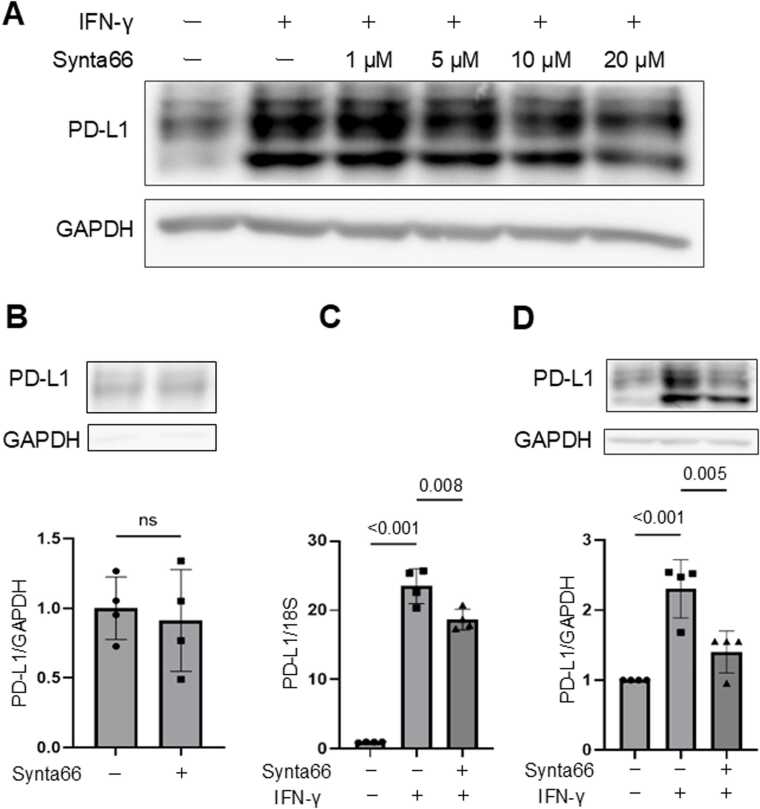
Pharmacological inhibition of SOCE suppresses IFN-γ–induced PD-L1 expression in OSCC cells. (A) HSC-3 cells were pretreated with the SOCE inhibitor Synta66 (1–20 μM) and stimulated with IFN-γ (100 ng/mL, 6 h). PD-L1 and GAPDH protein levels were analyzed by Western blot. PD-L1 is detected as multiple bands, which most likely reflect heterogeneous N-linked glycosylation and maturation states of the protein. (B) PD-L1 expression was examined in cells treated with Synta66 (20 μM) alone under unstimulated conditions.　(C) Cells were pretreated with Synta66 (20 μM, 3 h), followed by IFN-γ (100 ng/mL, 3 h). PD-L1 mRNA was assessed by RT-qPCR.　(D) Cells were pretreated with Synta66 (20 μM, 3 h), followed by IFN-γ (100 ng/mL, 6 h). PD-L1 protein levels were analyzed by Western blot. Quantitative data are shown as mean ± SEM from four independent biological replicates (n = 4). Exact p-values or significance levels (p < 0.001 or ns) are indicated in the figure. Representative Western blot images are shown.

Based on our proteomic findings that IFN-γ stimulation enriched calcium-related pathways, we next investigated the role of calcium influx through SOCE in PD-L1 regulation. SOCE regulates diverse physiological processes, including gene expression, secretion, and cell survival, and has been implicated in cancer cell proliferation and immune evasion [30]. Given the central role of IFN-γ in tumor immunity and its ability to induce interferon-stimulated genes including PD-L1, we investigated whether SOCE inhibition affects PD-L1 expression. HSC-3 cells were pretreated with the SOCE inhibitor Synta66 and subsequently stimulated with IFN-γ. PD-L1 protein expression, which was induced by IFN-γ, was suppressed by Synta66 in a dose-dependent manner (Fig. 2A). Notably, we confirmed in advance that Synta66 did not exhibit significant cytotoxicity against HSC-3 cells, as assessed by the CCK-8 assay (Fig. S1). In the absence of IFN-γ, Orai1 inhibition had minimal effect on basal PD-L1 expression (Fig. 2B). However, at a concentration of 20 μM-where suppression was most pronounced, IFN-γ-induced PD-L1 mRNA expression was significantly reduced (Fig. 2C), and this effect was also evident at the protein level (Fig. 2D). Importantly, consistent results were obtained in another OSCC cell line, OSC-19, in which Synta66 similarly attenuated IFN-γ-induced PD-L1 expression at both the protein and mRNA levels (Fig. S2). These data further support that SOCE inhibition broadly suppresses IFN-γ-driven PD-L1 induction across multiple OSCC cell lines. Together, these findings suggest that SOCE is an essential modulator of IFN-γ-induced PD-L1 expression. To verify that SOCE was functionally suppressed under both pharmacological and genetic inhibition conditions, intracellular Ca²⁺ dynamics were examined using Fluo 4-AM–based Ca²⁺ imaging following thapsigargin-induced ER Ca²⁺ depletion and extracellular Ca²⁺ re-addition (Fig. S3).

## Genetic suppression of SOCE components Orai1 attenuates IFN-γ-induced PD-L1 expression

**Fig. 3:**
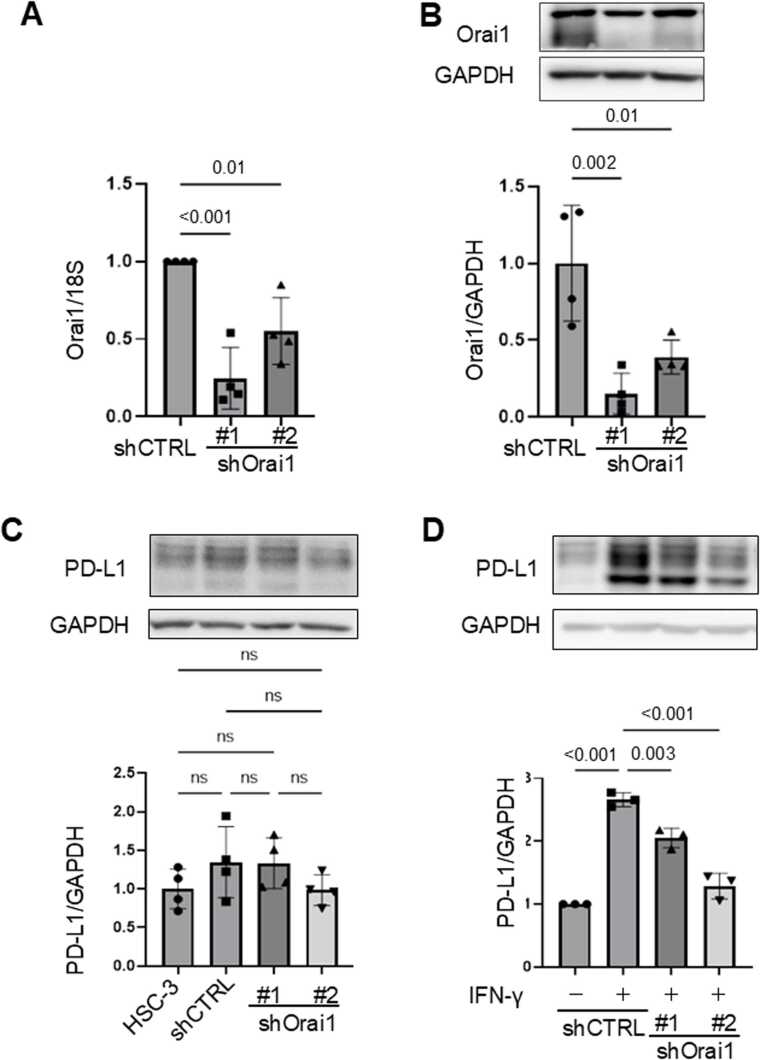
Genetic knockdown of Orai1 suppresses IFN-γ–induced PD-L1 expression in HSC-3 cells. (A, B) HSC-3 cells were transduced with two shRNAs targeting Orai1 (shOrai1#1, #2). Orai1 expression was analyzed by RT-qPCR (mRNA) and Western blot (protein). (C) PD-L1 protein expression was examined under basal conditions in Orai1 knockdown and control cells. (D) Cells were stimulated with IFN-γ (100 ng/mL, 6 h), and PD-L1 protein expression was analyzed by Western blot. Quantitative data in panels (A–C) are shown as mean ± SEM from four independent biological replicates (n = 4), whereas quantification in panel (D) was performed using three independent biological replicates (n = 3). Exact p-values or significance levels (p < 0.001 or ns) are indicated. Representative Western blot images are shown.

We next investigated the effect of genetic inhibition of Orai1, a key molecule involved in SOCE, on IFN-γ-induced PD-L1 expression. Knockdown of Orai1 was achieved using two independent shRNAs (shOrai1#1 and #2), and its efficiency was confirmed by RT-qPCR and Western blot analysis, both of which demonstrated significant reductions in Orai1 mRNA and protein levels (Fig. 3A, Fig. 3B). In the absence of IFN-γ, Orai1 knockdown had minimal impact on basal PD-L1 expression (Fig. 3C). However, while control cells (shCTRL) exhibited a marked increase in PD-L1 protein expression in response to IFN-γ stimulation, this induction was abrogated in both shOrai1#1 and shOrai1#2 knockdown cell clones (Fig. 3D). These findings indicate that Orai1 is essential for the upregulation of PD-L1 expression upon IFN-γ stimulation.

## Genetic suppression of STIM1, a core component of SOCE, attenuates IFN-γ-induced PD-L1 expression

**Fig. 4:**
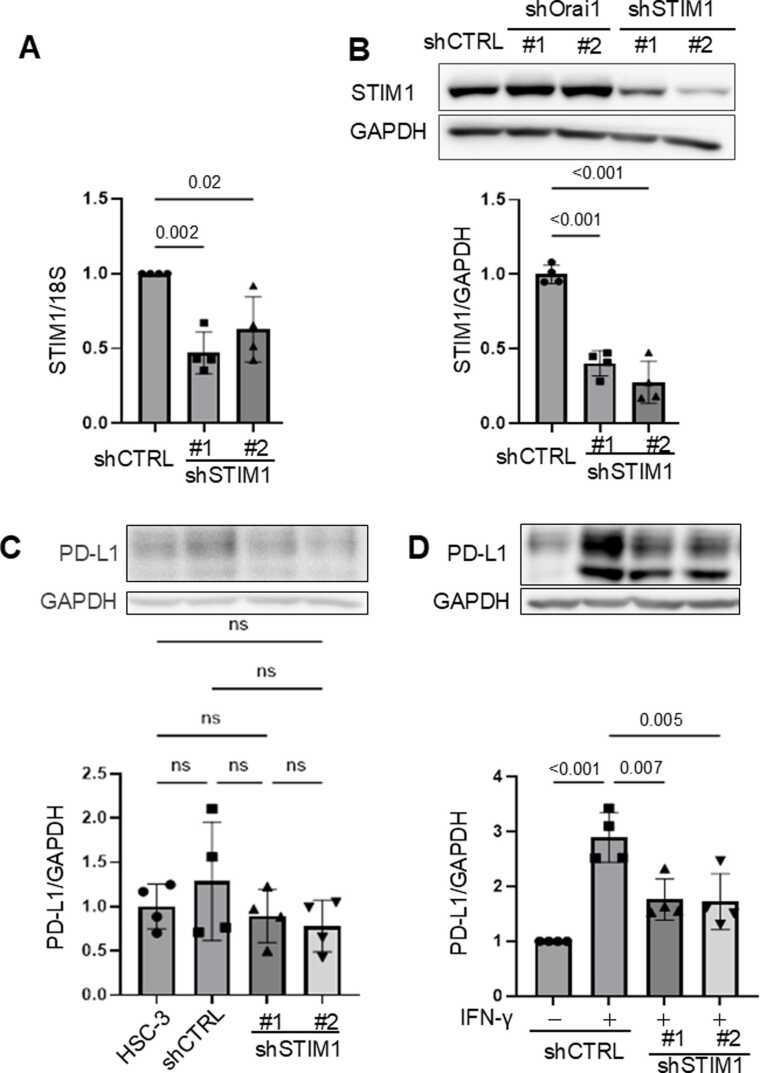
Genetic knockdown of STIM1 suppresses IFN-γ–induced PD-L1 expression in HSC-3 cells. (A, B) HSC-3 cells were transduced with two shRNAs targeting STIM1 (shSTIM1#1, #2). STIM1 expression was analyzed by RT-qPCR (A) and Western blot (B). For direct comparability under identical experimental conditions, lysates from shCTRL, shOrai1, and shSTIM1 cells were resolved on the same membrane; quantification and statistical analyses were restricted to comparisons between shCTRL and shSTIM1 groups. (C) PD-L1 protein expression was examined under basal conditions in STIM1 knockdown and control cells. (D) Cells were stimulated with IFN-γ (100 ng/mL, 6 h), and PD-L1 protein expression was analyzed by Western blot. Data are shown as mean ± SEM from four independent biological replicates (n = 4). Exact p-values or significance levels (p < 0.001 or ns) are indicated. Representative Western blot images are shown.

STIM1 is an ER calcium sensor that detects ER calcium depletion and activates Orai1 channels on the plasma membrane, thereby initiating SOCE [13], [31]. To evaluate the role of STIM1 in PD-L1 regulation, we assessed the knockdown efficiency of STIM1 using two independent shRNAs (shSTIM1#1 and #2), as performed for Orai1. Both RT-qPCR and Western blot analyses confirmed a significant reduction in STIM1 mRNA and protein levels (Fig. 4A, Fig. 4B). In the absence of IFN-γ, STIM1 knockdown did not alter basal PD-L1 expression (Fig. 4C). However, while control cells (shCTRL) showed a marked increase in PD-L1 protein expression upon IFN-γ stimulation, this induction was abrogated in both shSTIM1#1 and shSTIM1#2 knockdown cell clones (Fig. 4D). These results indicate that both Orai1 and STIM1 are essential for IFN-γ-induced PD-L1 protein expression, highlighting the critical role of SOCE in mediating this response in oral cancer cells.

## IFN‑γ‑induced PD‑L1 expression is regulated via CaMK2 and CaMKK2

In the context of calcium signaling downstream of SOCE, calmodulin-dependent kinases (CaMKs) are key mediators [32]. CaMKs are serine/threonine kinases activated by Ca²⁺/calmodulin. Among them, CaMK2 is directly activated downstream of SOCE-mediated Ca²⁺ influx and has been shown to regulate key cancer-related processes such as proliferation, migration, and immune modulation　[33], [34]. CaMKK2 functions upstream of other CaMKs and is also activated by SOCE, phosphorylating targets such as CaMK1 and AMP-activated protein kinase [35]. Recent studies have demonstrated that both CaMK2 and CaMKK2 mediate signaling pathways downstream of SOCE in various cell types, including cancer cells. This raises the possibility that these kinases are involved in the regulation of immune checkpoint molecules, including PD-L1 [12], [36].

**Fig. 5:**
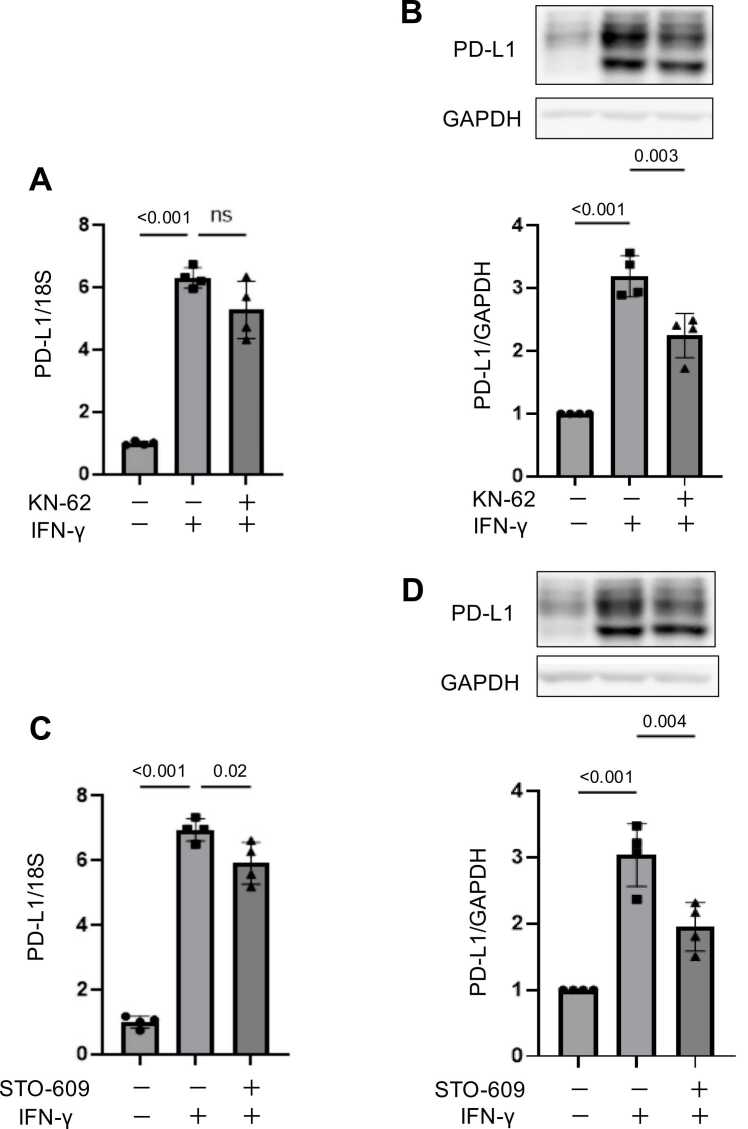
Inhibition of CaMK2 or CaMKK2 suppresses IFN-γ–induced PD-L1 expression in HSC-3 cells. (A, B) Cells were pretreated with the CaMK2 inhibitor KN-62 (10 μM, 60 min) and stimulated with IFN-γ (100 ng/mL) for 3 h (mRNA, RT-qPCR) or 6 h (protein, Western blot). (C, D) Cells were pretreated with the CaMKK2 inhibitor STO-609 (10 μM, 60 min) and stimulated with IFN-γ for 3 h (mRNA, RT-qPCR) or 6 h (protein, Western blot). Quantitative data are shown as mean ± SEM from four independent biological replicates (n = 4). Exact p-values or significance levels (p < 0.001 or ns) are indicated. Representative Western blot images are shown.

To elucidate the involvement of the downstream calmodulin-dependent kinase pathway following SOCE, we assessed the effect of pharmacologic inhibition using KN-62 (a CaMK2 inhibitor) and STO-609 (a CaMKK2 inhibitor) on IFN‑γ-induced PD‑L1 expression. Notably, we confirmed in advance that KN-62 and STO-609 did not exhibit significant cytotoxicity against HSC-3 cells, as assessed by the CCK-8 assay (Fig. S4, Fig. S5).　Upon treatment with KN‑62, RT‑qPCR and Western blot analysis revealed that IFN‑γ‑induced PD‑L1 mRNA levels showed a downward trend, and PD‑L1 protein expression was significantly suppressed (Fig. 5A, Fig. 5B), indicating a role for CaMK2 in mediating PD‑L1 induction. Similarly, STO‑609 significantly inhibited IFN‑γ‑induced PD‑L1 expression at both the mRNA and protein levels (Fig. 5C, Fig. 5D), indicating that the CaMKK2 pathway is also required for regulation of PD‑L1 expression. Taken together, these results suggest that IFN‑γ‑induced PD‑L1 expression is regulated through the activation of both CaMK2 and CaMKK2 downstream of SOCE-mediated calcium influx, highlighting a novel regulatory axis for immune checkpoint control in cancer cells.

**Genetic inhibition of Orai1 or STIM1 in oral cancer cells enhances CD8⁺ T cell–mediated antitumor activity**

To evaluate whether the reduced expression of PD-L1 following Orai1 or STIM1 knockdown affects tumor cell susceptibility to T cell–mediated cytotoxicity, we performed co-culture assays using HSC-3 oral squamous cell carcinoma cells and CD8⁺ T cells isolated from healthy donors. Notably, we confirmed by flow cytometry that CD8⁺ T cells were properly isolated and cultured (Fig. S6).

**Fig. 6:**
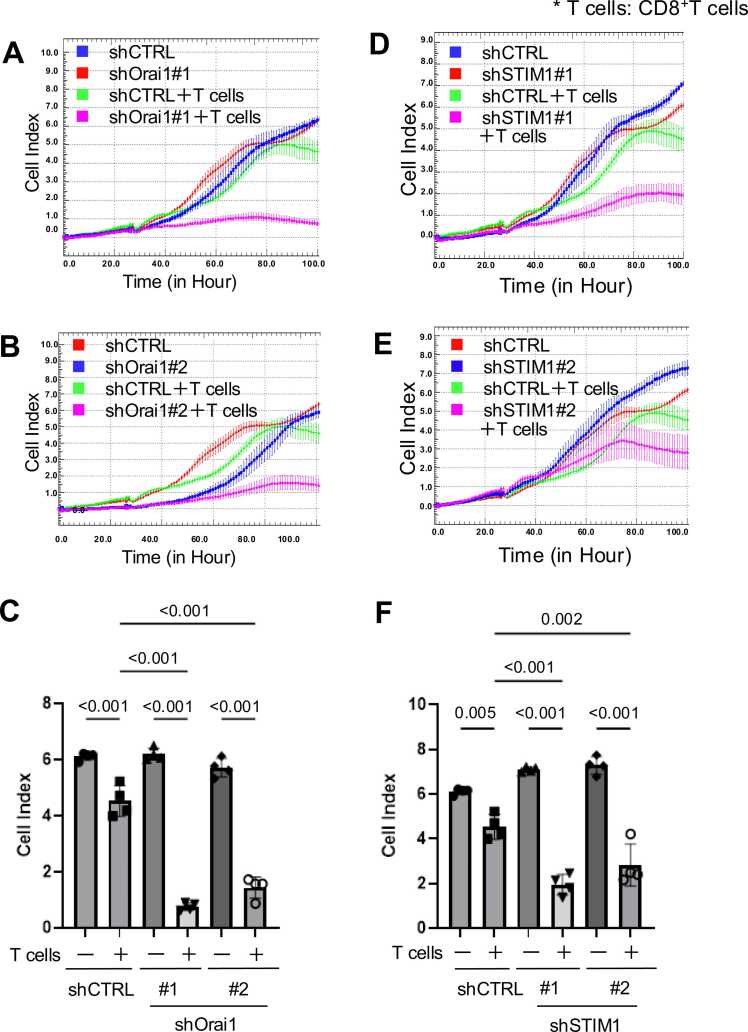
Knockdown of Orai1 or STIM1 enhances CD8⁺ T cell–mediated cytotoxicity against HSC-3 cells. (A–C) Control (shCTRL) or Orai1 knockdown (shOrai1#1, #2) HSC-3 cells were co-cultured with CD8⁺ T cells, and Cell Index was monitored using the xCELLigence system. Quantification at 100 h is shown.　(D–F) Control (shCTRL) or STIM1 knockdown (shSTIM1#1, #2) HSC-3 cells were analyzed under the same conditions. Quantification at 100 h is shown. Data are shown as mean ± SEM from four independent biological replicates (n = 4). Exact p-values or significance levels (p < 0.001) are indicated.

Using xCELLigence real-time cell analysis, we observed that the Cell Index of Orai1 knockdown cells (shOrai1#1 and #2) was significantly decreased during co-culture with CD8⁺ T cells, compared to control cells (shCTRL), indicating enhanced cytotoxic activity of CD8⁺ T cells toward Orai1-deficient tumor cells (Fig. 6A–C). In contrast, in the absence of CD8⁺ T cells, Orai1 knockdown did not affect cell viability (Fig. S7).

Similarly, STIM1 knockdown cells (shSTIM1#1 and #2) also exhibited a significant reduction in Cell Index during co-culture with CD8⁺ T cells, consistent with enhanced immune-mediated killing (Fig. 6D–F). No viability changes were also observed in STIM1 knockdown cells cultured without T cells (Fig. S7).

These findings suggest that Orai1 and STIM1 in oral cancer cells play critical roles in suppressing CD8⁺ T cell–mediated antitumor responses, likely through regulation of IFN-γ–induced PD-L1 expression. Thus, SOCE appears to modulate the tumor immune microenvironment by controlling PD-L1–dependent evasion from CD8⁺ T cell immunity (Fig. S8).

## Discussion

In this study, we have demonstrated for the first time that the SOCE pathway, centered on Orai1 and STIM1, is involved in the regulation of PD-L1 expression in response to IFN-γ stimulation in OSCC cells. Our proteomic analysis showed significant enrichment of calcium-related GO terms after IFN-γ stimulation, suggesting the involvement of Ca²⁺ signaling. Based on these findings, we investigated the regulatory mechanisms mediated by the SOCE components Orai1 and STIM1. Knockdown of Orai1 or STIM1, as well as treatment with the SOCE inhibitor Synta66, significantly suppressed IFN-γ-induced PD-L1 expression, whereas the basal PD-L1 expression under non-stimulated conditions remained largely unaffected. These results indicate that SOCE selectively regulates the upregulation of PD-L1 expression induced by IFN-γ. In our Western blot analyses, PD-L1 was detected as multiple bands, consistent with previous studies reporting heterogeneous N-linked glycosylation and maturation states of PD-L1 [37], [38]. The higher–molecular weight forms likely correspond to mature, fully glycosylated PD-L1 at the plasma membrane, whereas lower bands represent partially glycosylated immature species.

Previous studies have widely reported the involvement of signaling pathways such as Janus Kinase/Signal Transducer and Activator of Transcription 1 (JAK/STAT1) and Interferon Regulatory Factor 1 in the transcriptional induction of PD-L1 [39]. However, the direct involvement of calcium signaling in this process remains incompletely understood.

In our enrichment network analysis based on IFN-γ–stimulated proteomic data (Fig. 1D), GO terms related to calcium signaling, such as “regulation of calcium ion transmembrane transport,” were located in close proximity to “JAK/STAT signaling” and “Interferon signaling.” This spatial clustering suggests a potential functional association between calcium signaling and IFN-γ–responsive transcriptional networks involved in PD-L1 induction. These findings support the hypothesis that calcium signaling may contribute to the regulation of immune checkpoint molecules in coordination with canonical interferon signaling pathways.

To further explore the potential intersection between SOCE and canonical IFN-γ signaling, we assessed STAT1 phosphorylation (Tyr701) and IRF1 expression following SOCE inhibition. However, no reproducible changes were observed in either p-STAT1 or IRF1 levels under our experimental conditions. These findings suggest that SOCE may not directly modulate the upstream JAK/STAT1–IRF1 axis but instead influence PD-L1 expression through calcium-dependent mechanisms acting in parallel with or downstream of canonical IFN-γ signaling.

In addition to these signaling analyses, our Ca²⁺ imaging experiments further clarified the relationship between IFN-γ stimulation and SOCE activity. IFN-γ did not induce an acute increase in intracellular Ca²⁺ levels, which is consistent with previous studies indicating that IFN-γ is not a classical Ca²⁺-mobilizing cytokine. In contrast, thapsigargin-evoked assays demonstrated that Orai1 knockdown, STIM1 knockdown, and Synta66 treatment markedly suppressed SOCE-mediated Ca²⁺ influx. These findings indicate that IFN-γ does not activate SOCE acutely but rather relies on basal or sustained SOCE activity to support downstream transcriptional programs leading to PD-L1 induction. This mechanistic insight strengthens the conclusion that SOCE contributes to IFN-γ–induced PD-L1 regulation through Ca²⁺ homeostatic processes rather than rapid Ca²⁺ transients.

In the present study, both mRNA and protein expression levels of PD-L1 were suppressed by the SOCE inhibitor Synta66, and protein expression was also suppressed by knockdown of Orai1 and STIM1, suggesting that calcium influx via SOCE represents a novel regulatory mechanism. Although Synta66 has been reported to act on channels other than Orai1 [40], [41], the fact that similar results were obtained at the mRNA level and that consistent trends were observed with multiple inhibitors strongly supports the involvement of SOCE. Moreover, inhibition of CaMK2 (by KN-62) and CaMKK2 (by STO-609), both downstream of SOCE, also suppressed PD-L1 expression, indicating that the calmodulin-dependent kinase cascade downstream of SOCE is implicated in the regulation of PD-L1. Because KN-62 inhibits CaMK2 in an isoform-non-selective manner, the present findings reflect the involvement of overall CaMK2 activity rather than a specific CaMK2 isoform in the regulation of PD-L1 expression.

In addition, while our data indicate that SOCE inhibition suppresses PD-L1 at both mRNA and total protein levels, we cannot exclude the possibility that SOCE also affects PD-L1 stability through post-translational mechanisms. Previous studies have shown that IFN-γ-induced PD-L1 accumulation is modulated by ubiquitination, glycosylation, and proteasomal or lysosomal degradation. Therefore, SOCE blockade might accelerate PD-L1 turnover independently of transcriptional suppression. Future studies will be required to clarify whether SOCE signaling regulates PD-L1 degradation dynamics in tumor cells.

In addition, we examined whether shRNA-based inhibition of SOCE affects the phosphorylation levels of CaMK2 and CaMKK2. However, we did not observe consistent or reproducible changes under these conditions (Fig. S9). These findings suggest that, at least under our experimental settings, SOCE does not directly regulate the phosphorylation status of these kinases.

NFAT and CREB are representative Ca²⁺-dependent transcription factors that act downstream of Orai1-mediated calcium influx and are known to regulate cytokine and chemokine expression in various immune and tumor cells. Although the potential involvement of these transcription factors in SOCE-dependent PD-L1 regulation under IFN-γ stimulation is intriguing, it has not yet been thoroughly characterized.

In the present study, we first aimed to clarify the overall impact of SOCE-mediated Ca²⁺ signaling on PD-L1 expression, and we demonstrated that inhibition of the Orai1/STIM1 pathway consistently reduced IFN-γ–induced PD-L1 expression. This finding establishes a foundation for future investigation of downstream transcriptional regulators. Further studies will be required to determine whether NFAT and CREB are activated and contribute to PD-L1 transcription following SOCE activation.

Furthermore, cancer cells with knockdown of Orai1 or STIM1 showed increased sensitivity to cytotoxic activity in co-culture experiments with CD8⁺ T cells. This suggests that regulation of PD-L1 expression via SOCE may be involved in the mechanisms of tumor immune evasion. Although surface PD-L1 expression was not directly quantified by flow cytometry in this study, the consistent reduction in total PD-L1 protein levels observed in Western blot analyses, together with enhanced CD8⁺ T-cell–mediated cytotoxicity, indicates that the observed modulation of PD-L1 is functionally relevant. The combination of these findings supports that SOCE-dependent PD-L1 regulation affects the immune recognition of tumor cells. Optimization of flow cytometric conditions for live-cell surface PD-L1 staining, including antibody selection, incubation parameters, and gating strategies, would require additional methodological development. Therefore, we focused on establishing the mechanistic link between SOCE inhibition, PD-L1 suppression, and increased T-cell–mediated killing, which we consider to provide sufficient evidence for our conclusions.

With respect to other cancer types, it has been reported in a non-Small Cell Lung Cancer model that knockdown of Orai1 markedly suppresses the secretion of exosomal PD-L1, leading to reactivation of T-cell responses and inhibition of tumor progression [42]. Although our study focused on the regulation of cell-surface PD-L1 expression and did not directly examine the secretion of exosomal PD-L1, our findings provide novel insights into the potential involvement of the SOCE pathway in immune evasion through PD-L1 regulation. Additionally, a clinical study involving 327 gastric cancer cases showed that high expression of Orai1 and STIM1 was significantly correlated with tumor progression, recurrence rate, and mortality [43]. Furthermore, in our previous study using melanoma cell lines, we reported that metastatic melanoma cells exhibited a significantly higher amplitude of SOCE compared to primary melanoma cells and normal melanocytes [15]. These findings, together with our results, support the notion that SOCE is commonly involved in tumor immune evasion, indicating potential universality across different types of cancer.

ICI therapy is also drawing attention as a therapeutic option for oral cancer; however, considerable inter-individual variation in efficacy and therapeutic resistance remains a challenge [44]. Our results suggest that inhibition of the SOCE pathway can enhance cytotoxic T cell activity by reducing PD-L1 expression, and therefore, the combination of SOCE inhibition with ICI therapy could potentially improve clinical outcomes. In addition to tumor cells, immunosuppressive myeloid cells in the tumor microenvironment—such as tumor-associated macrophages, myeloid-derived suppressor cells (MDSCs), and dendritic cells—also express PD-L1 and contribute to the suppression of cytotoxic T-cell activity in OSCC [10]. Recent studies have shown that the SOCE pathway regulates cytokine secretion (e.g., IL-10, TGF-β) and chemokine expression (e.g., CCL2, CXCL8) in these myeloid cells [10], [45], [46]. Therefore, SOCE blockade may not only reduce PD-L1 expression in tumor cells but also influence the immunosuppressive characteristics of the tumor microenvironment. In this study, we mainly focused on the tumor cell–intrinsic regulation of PD-L1 by SOCE. Future investigations using tumor cell–specific and myeloid cell–specific Orai1/STIM1 inhibition models will be important to distinguish tumor-intrinsic and microenvironment-dependent effects of SOCE on immune suppression. Furthermore, since the expression of Orai1 correlates with PD-L1 expression in several types of cancer [42], SOCE components may also serve as potential biomarkers for predicting PD-L1 expression.

There are several limitations to this study. Although we did not perform siRNA-resistant re-expression or CRISPR knockout plus rescue experiments, potential off-target concerns were mitigated by the concordant results obtained through multiple independent approaches: pharmacological inhibition of SOCE (Synta66), RNAi-mediated knockdown of Orai1 and STIM1, and consistent suppression of PD-L1 expression and enhancement of CD8⁺ T-cell cytotoxicity. These mechanistically distinct interventions collectively support that the observed effects are on-target and specifically mediated through SOCE inhibition.

First, the inhibitors used (Synta66, KN-62, STO-609) may have off-target effects and are not entirely specific [47]. However, the fact that the results were consistent with those observed at the mRNA level, as well as the trends observed with multiple inhibitors, supports the reliability of our findings. Second, we did not directly measure SOCE activity by real-time intracellular Ca²⁺ influx analysis; thus, our evaluation was limited to indirect assessment through pharmacological and genetic interventions. Further studies, including correlation analyses using clinical specimens, evaluation of therapeutic efficacy in in vivo models, and detailed pharmacological characterization of calcium channel inhibitors, will be needed to advance clinical applications. Future studies involving validation using in vivo models and clinical specimens will be required to clarify the clinical significance and reproducibility of these findings.

In summary, our findings have elucidated a novel pathway in which SOCE and the downstream calmodulin-dependent kinase cascade regulate IFN-γ-induced PD-L1 expression. This study provides new insights into the mechanisms of tumor immune evasion and suggests the potential clinical utility of targeting SOCE for the enhancement of ICI therapy, as well as the development of SOCE components as biomarkers. A graphical summary of the proposed mechanism is provided (Fig. S8).

## Ethics statement

School of Medicine. Peripheral blood samples were collected from healthy adult volunteers without known underlying diseases, and written informed consent was obtained from all participants prior to blood collection, in accordance with the Declaration of Helsinki. In addition, this study was conducted in accordance with the regulations for recombinant DNA experiments, and the experimental protocol was approved by the Yokohama City University School of Medicine (Approval number: F-D-21–60–5).

-Approval of the research protocol by an Institutional Review Board: Approved (Yokohama City University School of Medicine; F230300040).

-Informed Consent: Obtained from all participants.

-Registry and the Registration No. of the study/trial: N/A.

-Animal Studies: N/A.

## Funding information

This study was supported in part by the 10.13039/501100001691Japan Society for the Promotion of Science (22K06928, 22K10154, 23K25180, 23K07483) and this research was also supported in part by the JST FOREST Program (Fusion Oriented REsearch for disruptive Science and Technology) of the Japan Science and Technology Agency (to M.U.).

## Consent for publication

All authors read and approved the final manuscript for publication.

## Author contributions

W.F., K.O., M.U. and Y.I. designed the study and prepared the manuscript. W.F., S.I., M.E. and A.N. conducted the pharmacological and molecular biological studies. W.F., K.N., C.H. and Y.I. performed bioinformatics analysis. W.F. and M. U. prepared the manuscript. U.Y., T.F., K.M. and Y.I. reviewed and edited the manuscript.

## Publisher's note

Springer Nature remains neutral with regard to jurisdictional claims in published maps and institutional affiliations.

## Declaration of Generative AI and AI-assisted technologies in the writing process

During the preparation of this study, the author used ChatGPT 4o for English writing and proofreading. After using this tool, the author carefully reviewed and edited the content as needed, and took full responsibility for the content of the published article.

## Declaration of Competing Interest

The authors declare the following financial interests/personal relationships which may be considered as potential competing interests: Masanari Umemura reports financial support and equipment, drugs, or supplies were provided by The Japan Society for the Promotion of Scienece. If there are other authors, they declare that they have no known competing financial interests or personal relationships that could have appeared to influence the work reported in this paper

## Data Availability

All the data and materials are available.
