# Supplementary material for: Orai1- and STIM1-mediated calcium signaling controls PD-L1 expression and modulates antitumor immunity in oral cancer
Source: J Physiol Sci. 2026 Feb 3;76(1):100063. doi: 10.1016/j.jphyss.2026.100063 (PMC12914183; doi:10.1016/j.jphyss.2026.100063)
Supplement: Supplementary material [file mmc1.docx]

**Figure S1.**

0.05

0.002

**(μM)**

**Cell viability (% of CTRL)**

**Supplementary Figure 1.**

Cell viability of HSC-3 cells treated with Synta66 at various concentrations (1–100 μM) for 24 hours, assessed using the CCK-8 assay. DMSO was used as a vehicle control, and values were normalized to the untreated control group (CTRL). Quantitative data are shown as mean ± SEM from four independent biological replicates (n = 4). Exact p-values are indicated in the figure; ns indicates not significant.

**Figure S2.**

**B**

**A**

**Synta66	− 　＋**

**PD-L1/GAPDH**

**−**

**PD-L1**

<0.001

**GAPDH**

0.05

<0.001

<0.001

**PD-L1/18S**

**−**

**＋**

**＋**

**＋**

**＋**

**−**

**−**

**IFN-γ**

**Synta66	− 　 ＋**

**IFN-γ**

**Supplementary Figure 2.**

(A) OSC-19 cells were pretreated with the SOCE inhibitor Synta66 (20 μM, 3 h) and stimulated with IFN-γ (100 ng/mL, 3 h). PD-L1 mRNA expression was analyzed by RT-qPCR.

(B) OSC-19 cells were pretreated with Synta66 (20 μM, 3 h) and stimulated with IFN-γ (100 ng/mL, 6 h). PD-L1 protein levels were analyzed by Western blot. PD-L1 is detected as multiple bands, which most likely reflect heterogeneous N-linked glycosylation and maturation states of the protein. Quantitative data are shown as mean ± SEM from four independent biological replicates (n = 4). Exact p-values and significance levels (p < 0.001) are indicated in the figure. Representative Western blot images are shown.

Bright-field

0 s

Synta66 (+)

**D**

Fluorescence (Fluo 4-AM)

0 s	200 s	700 s

Synta66 (−)

shCTRL

shSTIM1

shOrai1

shCTRL

Synta66 (–)

Synta66 (+)

**B**

**C**

**A**

**Figure S3.**

**Supplementary Figure 3.**

This figure shows intracellular Ca²⁺ dynamics measured by Fluo 4-AM imaging to assess store-operated calcium entry (SOCE) under pharmacological inhibition or genetic knockdown conditions.

A. Intracellular Ca²⁺ traces in HSC-3 cells treated with or without the SOCE inhibitor Synta66 (20 μM, 3 h).

B. Intracellular Ca²⁺ traces comparing shCTRL and shOrai1 cells.

C. Intracellular Ca²⁺ traces comparing shCTRL and shSTIM1 cells.

D. Representative bright-field and Fluo 4-AM fluorescence images of HSC-3 cells treated with or without Synta66 (20 μM, 3 h). Brightfield images at 0 s indicate cell presence in the imaged fields.

In panels A–C, cells were incubated under Ca²⁺-free conditions, treated with thapsigargin to deplete ER Ca²⁺ stores, and subsequently subjected to extracellular Ca²⁺ re-addition to evoke SOCE; traces represent mean ΔF/F₀ values calculated from 50 randomly selected cells per condition.

Representative images are shown for pharmacological inhibition conditions to illustrate Ca²⁺ responses under identical cellular background.

**Figure S4.**

0.002

0.05

**(μM)**

**Cell viability (% of CTRL)**

**Supplementary Figure 4.**

Cell viability of HSC-3 cells treated with KN-62 at various concentrations (1–100 μM) for 24 hours, assessed using the CCK-8 assay. DMSO was used as a vehicle control, and values were normalized to the untreated control group (CTRL). Quantitative data are shown as mean ± SEM from four independent biological replicates (n = 4). Exact p-values are indicated in the figure; ns indicates not significant.

**Figure S5.**

<0.001

0.009

**Cell viability (% of CTRL)**

**(μM)**

**Supplementary Figure 5.**
Cell viability of HSC-3 cells treated with STO-609 at various concentrations (1–100 μM) for 24 hours, assessed using the CCK-8 assay. DMSO was used as a vehicle control, and values were normalized to the untreated control group (CTRL). Quantitative data are shown as mean ± SEM from four independent biological replicates (n = 4). Exact p-values or significance levels (p < 0.001 or ns) are indicated in the figure.

**Figure S6.**

**CD4 (FITC)**

**CD4 (FITC)**

**CD4 (FITC)**

**CD4 (FITC)**

**CD8 (PE-Cy7)**

**CD8 + 7-AAD**

**CD8**

**F**

**7-AAD**

**E**

**7-AAD**

**7-AAD**

**CD4 Isotype Control**

**D**

**A**

**C**

**B**

**7-AAD**

**Unstained**

**CD4 + CD8**

**7-AAD**

**CD8 Isotype Control**

**7-AAD**

**CD8 (PE-Cy7)**

**Supplementary Figure 6.**

This figure shows the confirmation of negative selection and isolation of CD8⁺ T cells.

A. CD8 isotype control (PE-Cy7).

B. Dual staining of CD4 and CD8 with 7-AAD.

C. Unstained control.

D. CD4 isotype control (FITC).

E. CD8-stained cells analyzed along the CD4 axis.

F. CD8-stained cells analyzed with CD4 and 7-AAD to identify live CD8⁺ T cells.

**Figure S7.**

**Relative cell viability**

**#1	#2	#1	#2**

**shCTRL**

**shSTIM1**

**shOrai1**

**Supplementary Figure 7.**
Proliferation of HSC-3 cells stably expressing shCTRL, shOrai1 (#1 and #2), or shSTIM1 (#1 and #2) cultured under standard medium conditions for 24 hours, assessed using the CCK-8 assay. Quantitative data are shown as mean ± SEM from four independent biological replicates (n = 4). Exact p-values are indicated in the figure; ns indicates not significant.

**Figure S8.**

**Supplementary Figure 8.**

Proposed mechanism of Orai1/STIM1-mediated SOCE in PD-L1 regulation (Created in BioRender. Yamashita, E. (2025) https://BioRender.com/1cecmlp).

**B**

**A**

**IFN-γ**

　　 ー　 ー	＋　 ＋	＋	＋　 ＋	＋

C

**GAPDH**

**shCTRL**

**shOrai1**

**shCTRL**

**shOrai1**

5 min

15 min

CTRL

**p-CaMKK2 (Ser511)**

**CaMKK2**

C

30 min

**shCTRL**

**shOrai1**

**shOrai1**

**shCTRL**

**Figure S9.**

**Supplementary Figure 9.**

This figure shows the effects of genetic inhibition of SOCE on the phosphorylation status of CaMK2 and CaMKK2 in response to IFN-γ stimulation.

A. Immunoblot analysis of phosphorylated CaMK2 (Thr286), total CaMKII, and GAPDH in shCTRL and shOrai1 cells under unstimulated conditions and after IFN-γ stimulation for the indicated times.

B. Immunoblot analysis of phosphorylated CaMKK2 (Ser511), total CaMKK2, and GAPDH in shCTRL and shOrai1 cells under unstimulated conditions and after IFN-γ stimulation for the indicated times. Under these experimental conditions, genetic inhibition of SOCE by Orai1 knockdown did not produce consistent or reproducible changes in the phosphorylation levels of CaMKII or CaMKK2 following IFN-γ stimulation. Representative immunoblots from multiple independent experiments are shown.

**Figure S10.**

**A**

PD-L1

**B**

GAPDH

PD-L1

**C**

GAPDH

PD-L1

GAPDH

**Supplementary Figure 10.**
A. Original membrane image for Fig. 2A.

B. Original membrane image for Fig. 2B.

C. Original membrane image for Figure 2D.

**Figure S11.**

**Supplementary Figure 11.**
Original membrane image for Fig. S2B.

PD-L1

GAPDH

**Figure S12.**

PD-L1

Orai1

GAPDH

**B**

**C**

**A**

GAPDH

GAPDH

PD-L1

**Supplementary Figure 12.**
A. Original membrane image for Fig. 3B.

B. Original membrane image for Fig. 3C.

C. Original membrane image for Figure 3D.

**Figure S13.**

PD-L1

GAPDH

PD-L1

GAPDH

**A**

**B**

**C**

GAPDH

STIM1

**Supplementary Figure 13.**
A. Original membrane image for Fig. 4B.

B. Original membrane image for Fig. 4C.

C. Original membrane image for Figure 4D.

**Figure S14.**

**B**

**A**

PD-L1

PD-L1

GAPDH

GAPDH

PD-L1

PD-L1

GAPDH

GAPDH

**Supplementary Figure 14.**
A. Original membrane image for Fig. 5B.

B. Original membrane image for Fig. 5D.

GAPDH

GAPDH

GAPDH

**B**

GAPDH

p-CaMKK2

(Thr286)

CaMK2

GAPDH

CaMK2

GAPDH

p-CaMK2

(Thr286)

**A**

CaMK2

p-CaMK2

(Thr286)

**Figure S15.**

**Supplementary Figure 15.**
A. Original membrane image for Fig. S10A.

B. Original membrane image for Fig. S10B.

CaMK2

p-CaMK2

(Thr286)
